# Cognitive behavioral therapy in obsessive-compulsive disorder: A review

**DOI:** 10.1192/j.eurpsy.2021.1117

**Published:** 2021-08-13

**Authors:** D. Duarte

**Affiliations:** Departamento De Psiquiatria E Saúde Mental, Centro Hospitalar Universitário do Algarve, Estoi, Faro, Portugal

**Keywords:** Obsessive-Compulsive disorder, Cognitive Therapy Behavioral, psychotherapy

## Abstract

**Introduction:**

Obsessive-compulsive disorder (OCD) is characterized by the presence of obsessive thoughts and recurring compulsive acts. The prevalence is 1-3% in the general population. The treatment consists of Cognitive Behavioral Therapy (CBT) alone, or in combination with antidepressants.

**Objectives:**

Provide an overview of the elements of CBT in this disease and the techniques used.

**Methods:**

The authors did a non-systematic review in Pubmed with the words: “Obsessive-Compulsive Disorder” and “Cognitive Behavioral Therapy”.

**Results:**

According to the cognitive-behavioral model model, in this disease intrusive thoughts arise spontaneously, normally and universally, interrupting the normal flow of thought. They are usually neutral and form the basis for vulnerable people to develop obsessive problems. CBT uses techniques that aim to correct dysfunctional thoughts and beliefs, as well as behavioral techniques that aim to change compulsive behaviors. It aims to help people to come to the conclusion that the problem is not in the intrusive thoughts, but in the meaning they attribute to them, and in the various strategies they adopt to try to control them. It basically follows the following steps - patient assessment, through one or more semi-structured interviews; initial phase that includes the assessment of motivation for treatment and psychoeducation; intermediate phase, with the continuation of monitored exercises and reinforcement of cognitive and behavioral techniques; discharge, with discharge preparation working on relapse prevention and maintenance therapy.

**Conclusions:**

Currently, CBT is considered the first-line therapy for the treatment of this disorder, however some patients are still refractory, and very little is known about its predictors of response.

